# Physical Environmental Correlates of Domain-Specific Sedentary Behaviours across Five European Regions (the SPOTLIGHT Project)

**DOI:** 10.1371/journal.pone.0164812

**Published:** 2016-10-14

**Authors:** Sofie Compernolle, Katrien De Cocker, Célina Roda, Jean-Michel Oppert, Joreintje D. Mackenbach, Jeroen Lakerveld, Ketevan Glonti, Helga Bardos, Harry Rutter, Greet Cardon, Ilse De Bourdeaudhuij, WP3 SPOTLIGHT group

**Affiliations:** 1 Department of Movement and Sport Sciences, Faculty of Medicine and Health Sciences, Ghent University, Ghent, Belgium; 2 Research Foundation Flanders (FWO), Brussels, Belgium; 3 Equipe de Recherche en Epidémiologie Nutritionnelle (EREN), Université Paris 13, Centre de Recherche en Epidémiologie et Statistiques, Inserm (U1153), Inra (U1125), Cnam, COMUE Sorbonne Paris Cité, Bobigny, France; 4 Université Pierre et Marie Curie-Paris 6, Department of Nutrition Pitié-Salpêtrière Hospital (AP-HP), Centre for Research on Human Nutrition Ile-de-France (CRNH IdF), Institute of Cardiometabolism and Nutrition (ICAN), Paris, France; 5 Department of Epidemiology and Biostatistics, EMGO Institute for Health and Care Research, VU Medical Center, Amsterdam, the Netherlands; 6 ECOHOST - The Centre for Health and Social Change, London School of Hygiene and Tropical Medicine, London, United Kingdom; 7 Department of Preventive Medicine, Faculty of Public Health, University of Debrecen, Debrecen, Hungary; Vanderbilt University, UNITED STATES

## Abstract

**Background:**

The relation between neighbourhood environmental factors and domain-specific sedentary behaviours among adults remains unclear. This study firstly aims to examine the association of perceived and objectively measured neighbourhood safety, aesthetics, destinations and functionality with transport-related, work-related and leisure-time sedentary behaviour. Secondly, the study aims to assess whether these associations are moderated by age, gender or educational level.

**Methods:**

In 60 randomly sampled neighbourhoods from 5 urban regions in Europe (Ghent and suburbs, Paris and inner suburbs, Budapest and suburbs, the Randstad, and Greater London), a virtual audit with Google Street View was performed to assess environmental characteristics. A total of 5,205 adult inhabitants of these neighbourhoods reported socio-demographic characteristics, sedentary behaviours, and neighbourhood perceptions in an online survey. Generalized linear mixed models were conducted to examine associations between physical environmental neighbourhood factors and sedentary behaviours. Interaction terms were added to test the moderating role of individual-level socio-demographic variables.

**Results:**

Lower levels of leisure-time sedentary behaviour (i.e. all leisure activities except television viewing and computer use) were observed among adults who perceived greater numbers of destinations such as supermarkets, recreational facilities, or restaurants in their neighbourhood, and among adults who lived in a neighbourhood with more objectively measured aesthetic features, such as trees, water areas or public parks. Lower levels of work-related sedentary behaviour were observed among adults who perceived less aesthetic features in their neighbourhood, and among adults who lived in a neighbourhood with less objectively measured destinations. Both age, gender and educational level moderated the associations between neighbourhood environmental factors and sedentary behaviours.

**Conclusion:**

Preliminary evidence was found for associations between neighbourhood environmental factors and domain-specific sedentary behaviours among adults. However, these associations varied according to objective or subjective environmental measures. More research is needed to confirm and clarify the associations.

## Background

High levels of sedentary behaviour, which can be defined as any waking activity characterized by an energy expenditure ≤1.5 metabolic equivalents (METs) while being in a sitting or reclining posture [1], are an important risk factor for numerous adverse health outcomes in adults [2–6]. Although some uncertainty remains [7], there is a growing consensus that the risks associated with prolonged sedentary behaviour remain even if individuals meet the public health recommendations for physical activity [4,8,9].

As postulated by ecological models of health behaviours [10,11], determinants of sedentary behaviour can be broadly categorized as individual or environmental level determinants [10,12]. Research on individual level determinants has indicated that several socio-demographic factors appear to influence adults’ sedentary behaviour [13]. However, these determinants are rarely modifiable, and therefore less relevant for the design of interventions by public health practitioners and community decision makers [14]. Consequently, efforts should be made to identify modifiable, upstream determinants, such as physical environment factors [15]. These physical environmental factors include the availability of sedentary behaviour opportunities at different levels (micro, meso, macro) [16,17]. Micro-level factors, such as the quantity of screens within the home, have been extensively studied [18]. But, understanding determinants at the meso or neighbourhood level also requires attention, as small changes in the physical neighbourhood environment may have a considerable public health impact, given the broad reach and long-term exposure of individuals to their neighbourhood environment [11,19].

Based on the framework of Pikora and colleagues [20], potential physical environmental neighbourhood factors of importance to health behaviours can be allocated to one of the following key constructs: functionality, safety, aesthetics, and destinations. Each of these constructs may be important for specific domains of sedentary behaviour [12,21,22]. In general, three domains of sedentary behaviour can be distinguished, including leisure-time (e.g. television viewing, computer use), transportation and occupation [21,22]. One might hypothesize that residents from safe, functional and attractive neighbourhoods with many local facilities will be less likely to spend time indoors watching television or using a computer. They may prefer travelling by bicycle or on foot, instead of by bus, tram or car, resulting in less transport-related sedentary behaviour [10]. Nevertheless, previous research has failed to find consistent evidence to support these hypotheses [23]. For example, of the four studies examining the association of neighbourhood safety with leisure-time of transport-related sedentary behaviour, one found a significant association in the expected direction (i.e. safer neighbourhoods are inversely associated with sedentary behaviours) [24], two studies did not find a significant association [25,26], and one study found a significant association in the unexpected direction [27]. These mixed results can partly be attributed to differences in measurement methods. Some studies used perceived physical environmental factors (e.g. [25,28]), whereas other studies used objectively measured physical environmental factors (e.g. [29–31]). Perceived physical environmental factors refers to the perceptions of residents, and are generally obtained from interviews or self-administered questionnaires. Objectively measured physical environmental factors are derived from systematic observations or calculated based on existing spatial data using geographic information systems [32]. As there is a clear mismatch between these two measurement methods [32–36], it is important to combine perceived and objectively measured physical environmental qualities to better understand the potential impact of the physical environment on sedentary behaviours [32]. Another explanation may be the lack of control of other variables. For example, residents from an attractive neighbourhood will not be motivated to reduce their transport-related sedentary behaviour if they do not perceive their neighbourhood as safe for active transport. A final explanation for the mixed results might be differences in demographic characteristics between study samples [12,17,23]. For example, physical environmental neighbourhood factors may be more important for older adults, as they are more susceptible to physical barriers, such as long distances, because of functional limitations [37], or for low SES adults, who may be more constrained to their own neighbourhood environment due to a lack of financial resources for travel outside their neighbourhood [38]. It is thus important to examine the moderating effects of these socio-demographic factors.

In contrast to leisure-time and transport-related sedentary behaviour, physical environmental neighbourhood correlates of work-related sedentary behaviour have not yet been investigated. This appears to be an important domain as adults spend a large amount of time sitting at work [39,40]. Work-related sedentary behaviour may be affected by physical environmental neighbourhood factors—even if the work is not situated within one’s neighbourhood [41,42]. It has been proposed that residents of attractive, functional and safe neighbourhoods with many local facilities might compensate for their lower transport-related and leisure-time sedentary behaviour by increasing their work-related sedentary behaviour. This could also explain the lack of consistent associations between physical environmental neighbourhood factors and total sedentary behaviour [23].

The first aim of this study was to examine the association of both perceived and objectively measured physical environmental neighbourhood factors with several domains of sedentary behaviour, including transport, occupation and leisure-time (watching television, using a computer at home, and other leisure-time activities), as well as with total sedentary behaviour. Based on previous studies, it was hypothesized that living in a safe, functional and attractive neighbourhood with many local facilities would be associated with less transport-related sedentary behaviour, less leisure-time sedentary behaviour and more work-related sedentary behaviour. The second aim was to identify socio-demographic variations within these associations by assessing the moderating effect of age, gender and educational level.

## Methods

### Procedure

This cross-sectional study was part of the European SPOTLIGHT (Sustainable Prevention of Obesity Through Integrated Strategies) project, which was designed to gain insight into the broad range of overweight and obesity-related correlates in adults [43]. The study was conducted in five urban regions: Ghent and suburbs (Belgium), Paris and inner suburbs (France), Budapest and suburbs (Hungary), the Randstad (a conurbation including the cities Amsterdam, Rotterdam, the Hague and Utrecht in the Netherlands) and Greater London (United Kingdom). Sampling of neighbourhoods and recruitment of participants has been described in detail elsewhere [44]. Neighbourhood sampling was based on a combination of residential density and SES data at the neighbourhood level. This resulted in four types of neighbourhoods: low SES/ low residential density, low SES/ high residential density, high SES/ low residential density and high SES/ high residential density. In each country, three neighbourhoods of each neighbourhood type were randomly sampled (i.e.12 neighbourhoods per country, 60 neighbourhoods in total). Subsequently, a random sample of adult inhabitants in each neighbourhood was invited to participate in an online survey. The survey contained questions on demographics, neighbourhood perceptions, social environmental factors, health, obesity-related behaviours (e.g. dietary habits, sedentary behaviours, and physical activity), motivations and barriers for obesity-related behaviours, and weight and height. A total of 6,037 (out of 55,893) individuals participated in the study between February and September 2014. The overall response rate was 10.8%, varying from 8.9% in low SES/high residential density neighbourhoods to 12.7% in high SES/low residential density neighbourhoods [44]. A total of 832 participants were excluded from the present analyses because they could not be geolocalized or because their neighbourhood was not covered by Google Street View (which was used for the objective measurements), resulting in a total of 5,205 participants. The study was approved by the corresponding local ethics committees of participating countries and all participants provided informed consent by ticking the following two boxes: 1) I declare that I have read the information letter and information sheet. I have had the opportunity to ask questions about the study if I wanted to, and have received satisfactory answers to questions, and any additional details requested, and 2) I declare that I agree to participate in the study. Participants who did not tick previous mentioned boxes, were not able to fill out the questionnaire.

### Measures

#### Self-reported sedentary behaviour

Domain-specific sedentary behaviour was measured using the Marshall questionnaire [45]. In this questionnaire, domain-specific sedentary time was estimated by asking the average number of hours and minutes participants’ spent sedentary during transport, work, television viewing, computer use at home and other leisure-time activities on both weekdays and weekend days during the last seven days [45]. Leisure-time activities include reading, socializing, going to a movie, etc. The Marshall questionnaire has acceptable reliability and validity, with the highest validity coefficients found for sitting time at work and using a computer at home (r = 0.69–0.74). Lowest validity coefficients were found for other leisure-time and transport-related sedentary behaviour during weekend-days (r = 0.15–0.42). As the use of computer tablets is becoming increasingly prevalent, ‘tablets’ was added to the list of options alongside television and computer. Total weekly domain-specific sedentary behaviour was estimated by summing the weekday (multiplied by five) and weekend day (multiplied by two) hours. This sum was divided by seven to express average domain-specific sedentary behaviour in hours/day. All domain-specific sedentary behaviours were summed to estimate total sedentary behaviour per day.

#### Physical environmental neighbourhood factors

Objectively measured physical environmental factors within participants’ neighbourhoods were assessed using the SPOTLIGHT virtual audit tool (S-VAT) [46]. The S-VAT has been shown to be a reliable and valid remote sensing tool to assess obesogenic environmental characteristics [46]. The S-VAT initially contained 42 items, grouped into eight domains: walking (six items), cycling (eight items), public transport (two items), aesthetics (nine items), land use mix (three items), grocery stores (five items), food outlets (six items) and recreational facility-related items (three items). All items were assessed in the 4,486 street segments (defined as a part of the street between two intersections with a minimum length of 50 meters and a maximum length of 300 meters) within the 59 selected neighbourhoods (one Hungarian neighbourhood was not covered by Google Street View at the time of the virtual audit) by trained researchers of the SPOTLIGHT project team. Street segment level data were aggregated to the neighbourhood level by taking the percentage of street segments with each feature in the neighbourhood [47]. Items from the S-VAT that were included in the current analyses are presented in Table 1.

**Table 1:** Description of the objectively measured and perceived physical environmental neighbourhood constructs.

Construct (Cronbach’s alpha)	Items
**Objectively measured physical environmental neighbourhood (n = 59 neighbourhoods)**	
**Safety (0.57)**	
	% streets with pedestrian crossings
	% streets with traffic calming devices
	% streets with bicycle lanes
	% streets with traffic lights
	% streets with well-maintained sidewalks
**Aesthetics (0.60)**	
	% streets with green or water areas
	% streets with a public park
	% streets with residential gardens
	% streets with trees
	% streets with good condition residential buildings
	% streets without abandoned or vacant buildings
**Destinations (0.79)**	
	% streets with tram or bus stops present
	% streets with supermarkets, local shops or convenience shops
	% streets with restaurants, fast food restaurants or take away restaurants
	% streets with café/bar
	% streets with public park or recreational facilities
**Functionality (0.66)**	
	% streets with sidewalk present
	% streets with good maintained sidewalks
	% streets with traffic calming devices
	% streets with speed limit of 30km/h or less
	% streets with bicycle lanes
	% streets with bus or tram stops
**Perceived physical environmental neighbourhood (n = 5,205 participants)**	
**Perceived safety (0.45)**	
	special cycle lanes present in the neighbourhood
	not a lot of busy traffic in the neighbourhood
	sufficient pedestrian crossings to cross busy roads
	traffic is usually slow in the neighbourhood
	crime levels are low in the neighbourhood
**Perceived aesthetics (0.64)**	
	play areas in the neighbourhood are well maintained
	the environment is pleasant to walk/cycle in
	neighbourhood generally free from rubbish, litter and graffiti
**Perceived destinations (0.77)**	
	supermarkets present in neighbourhood
	local shop present in neighbourhood
	restaurant/café/bar present in neighbourhood
	fast food restaurant / take away present in neighbourhood
	open recreation area present in neighbourhood
	leisure facility present in neighbourhood
**Perceived functionality (0.72)**	
	special cycle lanes are present in the neighbourhood
	cycle paths in the neighbourhood are well maintained
	sidewalks in the neighbourhood are well maintained
	sufficient pedestrian crossings to cross busy roads
	traffic is usually slow in the neighbourhood
	there is a choice of routes in the neighbourhood

SD = standard deviation

Perceived physical environmental neighbourhood factors were assessed using the online survey. Participants were asked about physical environmental characteristics in what they perceived as their neighbourhood. The presence of facilities was asked using the following question: ‘Which of the following facilities (supermarket, local shop, restaurant/bar/café, fast-food restaurant/take away, open recreation area, leisure-time physical activity facility) are present in your neighbourhood? Other perceived physical environmental factors were assessed using items from the reliable and validated Assessing Levels of Physical Activity (ALPHA) questionnaire [48] and from the Multi Ethnic Study of Atherosclerosis (MESA) survey [49]. The items covered different aspects of the neighbourhood environment (see Table 1) and were assessed with a 5-point Likert scale ranging from 1 (totally disagree) to 5 (totally agree). The coding of some items was reversed for the analyses to ensure consistency in terms of directions of effects—i.e. so that a higher score indicated a more positive perception of the neighbourhood environment.

Both objectively measured and perceived physical environmental neighbourhood factors were grouped into four constructs: 1) functionality, 2) safety, 3) aesthetics, and 4) destinations. Grouping was based on the framework of Pikora et al. [20] using factor analysis (see Table 1). For each construct, the average of the included items was calculated. Internal consistency was checked for each construct. Cronbach’s alphas ranged from 0.57 to 0.79 for the objectively measured physical environmental constructs, and from 0.45 to 0.77 for the perceived physical environmental constructs.

#### Socio-demographic factors, body mass index (BMI) and physical activity

The following socio-demographic factors were assessed: age, gender, educational level (highest qualification achieved), employment status (currently employed, currently not employed), and household composition (number of adults and children in the household). To examine their moderating effects, both age and educational level were dichotomized. Age was dichotomised into adults (< 65 years) and older adults (≥ 65 years), as 65 years is generally the age of retirement in Europe, which often tends to be accompanied by a considerable change in sedentary behaviours [50]. Educational level was dichotomised into lower (no tertiary education) and higher educational level (tertiary education). Body mass index (BMI) was calculated by dividing self-reported weight in kilograms by the square of the self-reported height in meters. Moderate-to-vigorous leisure-time and transport-related physical activity in the last seven days was measured using corresponding items from the long version of the International Physical Activity Questionnaire [51].

### Data analysis

Descriptive statistics were analysed using SPSS 21, while the other statistical analyses were performed with R software, version 3.1.2. As the majority of the dependent variables (i.e. domain-specific and total sedentary behaviours) were non-normally distributed, generalized linear mixed models (GLMMs) were used to examine the first aim (i.e. testing the main associations of perceived and objectively measured neighbourhood factors with the domain-specific sedentary behaviours and total sedentary behaviour) [52]. The following domain-specific sedentary behaviours were included in the analyses: transport-related sedentary behaviour, work-related sedentary behaviour and leisure-time sedentary behaviour. Leisure-time sedentary behaviour was divided into television time, computer use, and other leisure-time sedentary behaviour, as previous studies indicated that correlates differ between those behaviours [53,54]. Different types of GLMMs are available, depending on the variance and link functions. The most appropriate variance (i.e. Gaussian, Gamma, and Logistic) and link functions (i.e. Identity and Log) of the GLMMs were selected based on Akaike’s Information Criterion (AIC) (i.e. models with the lowest AIC value represent the best model fit). Specifically, the associations between the neighbourhood factors (objective, perceived) and sedentary behaviour during transport, television viewing, and computer use at home were assessed using a GLMM with Gamma variance and identity link function. The association of the objectively measured and perceived neighbourhood factors with work-related sedentary behaviour and total sedentary behaviour was evaluated using a GLMM with Gaussian variance and identity link function. For leisure-time sedentary behaviour, Hurdle models were fitted, given the excessive number of zeros (24%). Hurdle models consist of two parts. First, a logistic regression model was fitted to estimate the relationship between physical environmental neighbourhood factors and the odds of participation in any other leisure-time sedentary activities. Second, a gamma regression model with log link function was fitted to estimate the relationship with the amount of other leisure-time sedentary activities among those participants that reported performing them. For each dependent variable, a random intercept variable was added to the model to account for clustering at neighbourhood level. Concretely, two separate regression models were fitted for each sedentary behaviour: one including all perceived physical environmental factors, and one including all objectively measured physical environmental factors. For the objectively measured neighbourhood factors, variance inflation factors (VIF) ranged from 1.51 to 2.90, and for perceived neighbourhood factors, VIF ranged from 1.03 to 2.75, revealing no multicollinearity [55]. The models initially included all socio-demographic variables, BMI and moderate-to-vigorous physical activity. Then a backward selection procedure, based on the AIC, was used to eliminate the covariates that did not improve the model fit. This implies that the final models only included the following covariates: age, gender, educational level, BMI and neighbourhood type and country. To examine the second aim (i.e. testing the moderating effects of age, gender and educational level), interaction terms (i.e. moderator x perceived or objectively measured physical environmental factor) were added to the twelve final models. In case of significant moderating effects, analyses were stratified by the factor in question to interpret the direction of the interactions. Statistical significance was set at p ≤ 0.05.

## Results

### Participant characteristics

Socio-demographic characteristics, descriptive statistics of BMI, sedentary behaviours and moderate-to-vigorous physical activity, as well as objectively measured and perceived physical environmental neighbourhood factors are shown in Table 2.

**Table 2:** Socio-demographic sample characteristics, sedentary behaviours and objectively measured/perceived physical environmental neighbourhood factors.

Variable		Total sample (n = 5,205)
**Socio-demographic characteristics**		
Gender (%)	Men	44.7
	Women	55.3
Age (years), mean (SD)		52.2 (16.3)
Educational level (%)	No tertiary education	45.9
	Tertiary education (college or university)	54.1
Employment status (%)	Unemployed/retired	17.8
	Employed	82.2
Household composition (%)	One-person household	22.8
	Two-person household	39.6
	Three-or more-person household	37.6
**Body mass index (kg/m**^**2**^**), mean (SD)**		25.2 (4.5)
**Sedentary behaviours**		
Total sedentary behaviour (hours/day), mean (SD)		8.90 (3.70)
Transport-related sedentary behaviour (hours/day), mean (SD)		1.38 (1.47)
Work-related sedentary behaviour (hours/day), mean (SD)^1^		4.29 (2.56)
Television time (hours/day), mean (SD)		2.62 (2.07)
Computer time at home (hours/day), mean (SD)		1.91 (1.87)
Other leisure-time sedentary behaviour (hours/day), mean (SD)		1.48 (1.65)
**Moderate-to-vigorous physical activity (hours/day), mean (SD)**		0.59 (0.84)
**Objectively measured physical environmental neighbourhood factors**		
Safety, median (Q1, Q3)		0.27 (0.13, 0.30) ^a^
Aesthetics, median (Q1, Q3)		0.68 (0.55, 0.76) ^a^
Destinations, median (Q1, Q3)		0.06 (0.04, 0.08) ^a^
Functionality, median (Q1, Q3)		0.34 (0.25, 0.52) ^a^
**Perceived physical environmental neighbourhood factors**		
Safety, median (Q1, Q3)		3.20 (2.80, 3.60) ^b^
Aesthetics, median (Q1, Q3)		3.67 (3.00, 4.00) ^b^
Destinations, median (Q1, Q3)		0.83 (0.67, 1.00) ^c^
Functionality, median (Q1, Q3)		3.50 (3.00, 4.00) ^b^

SD = Standard deviation

Q1 = quartile 1, Q3 = quartile 3

^a^ These numbers represents proportions (see Table 1)

^b^ Items of these constructs were assessed with a 5-point Likert scale ranging from 1 (totally disagree) to 5 (totally agree) (see Table 1)

^c^ Items of this construct were assessed with yes (= 1) or no (= 0) (see Table 1).

^1^ Only for those who are currently employed (n = 2855).

### Main associations of objectively measured and perceived physical environmental neighbourhood factors with sedentary behaviours

Table 3 presents the results of the main associations adjusted for age, gender, educational level, BMI, neighbourhood type and country. Significant associations were found for the objectively measured physical environmental neighbourhood factors of aesthetics and destinations. Living in a neighbourhood with better aesthetics was associated with being less likely to engage in leisure-time sedentary behaviour (i.e. all leisure activities except television viewing and computer use). Living in a neighbourhood with more destinations was associated with more work-related sedentary behaviour. More specifically, a 10% increase in destinations was associated with an 1.07 hour increase in work-related sedentary behaviour per day. Significant associations were found for the perceived physical environmental neighbourhood factors of aesthetics and destinations. Living in a neighbourhood with a higher score for perceived aesthetics was associated with more total and work-related sedentary behaviour. For example, a one-unit increase in perceived aesthetics (on a scale of 5) was related to spending 0.19 hour (11.4 minutes) more sedentary per day. The score for perceived destinations was negatively associated with leisure-time sedentary behaviour, i.e. a one-unit decrease in perceived destinations was associated with being 1.20 (1/0.83) times more likely to have spent time in leisure-time sedentary behaviour during the last seven days.

**Table 3:** Association of physical environmental neighbourhood factors with domain-specific sedentary behaviours.

	Total sedentary behaviour	Transport-related sedentary behaviour	Work-related sedentary behaviour	Television time	Computer time	Other leisure-time sedentary behaviour	
	Gaussian model	Gamma model	Gaussian model	Gamma model	Gamma model	Logistic model^1^	Gamma model^2^
	b (S.E.) p	b (S.E.) p	b (S.E.) p	b (S.E.) p	b (S.E.) p	OR (95% C.I.)	Exp b (95% C.I.)
**Objectively measured physical environment**							
Traffic safety	-0.82 (1.52) 0.59	-0.59 (0.71) 0.40	-2.35 (1.62) 0.15	0.43 (0.86) 0.61	0.46 (0.87) 0.60	0.64 (0.06, 1.97)	0.99 (0.08, 11.80)
Aesthetics	0.23 (0.71) 0.75	0.29 (0.32) 0.36	0.13 (0.71) 0.86	0.18 (0.31) 0.56	0.01 (0.41) 0.98	**0.12 (0.03, 0.41)**	1.00 (0.32, 3.11)
Destinations	8.01 (5.22) 0.13	1.47 (2.34) 0.53	**10.71 (5.31) 0.04**	-3.98 (2.53) 0.11	0.58 (3.14) 0.06	26.78 (0.00, 12.48)	1.00 (0.00, 4252.58)
Functionality	-0.41 (1.38) 0.77	0.14 (0.67) 0.83	0.43 (1.49) 0.77	-0.20 (0.81) 0.80	-0.69 (0.80) 0.39	0.34 (0.04, 1.10)	1.00 (0.08, 11.80)
**Perceived physical environment**							
Safety	-0.13 (0.17) 0.45	-0.08 (0.06) 0.17	0.03 (0.16) 0.86	-0.04 (0.09) 0.69	-0.05 (0.08) 0.59	0.93 (0.77, 1.22)	1.04 (0.81, 1.34)
Aesthetics	**0.19 (0.10) 0.05**	-0.03 (0.04) 0.37	**0.20 (0.10) 0.04**	-0.10 (0.06) 0.10	0.02 (0.05) 0.65	1.05 (0.90, 1.24)	0.98 (0.84, 1.14)
Destinations	-0.02 (0.08) 0.81	-0.02 (0.03) 0.57	-0.04 (0.07) 0.50	-0.01 (0.05) 0.77	0.02 (0.04) 0.60	**0.83 (0.74, 0.93)**	1.00 (0.89, 1.12)
Functionality	0.01 (0.16) 0.93	0.09 (0.05) 0.09	-0.14 (0.15) 0.35	0.04 (0.09) 0.67	0.00 (0.08) 0.96	1.30 (1.00, 1.67)	0.97 (0.77, 1.22)

Significant values are indicated in bold.

OR = odds ratio, 95% C.I. = confidence interval at 95%, S.E. = standard error

^1^ The logistic model estimates the associations between the independent variables and the odds of having time spent sedentary during other leisure activities in the last 7 days.

^2^ The gamma model estimates the associations between the independent variables and the amount of other leisure-time sedentary behaviours in the last 7 days.

All analyses were adjusted for age, gender, educational level, BMI, neighbourhood type and country.

All b-values represent the increase in (domain-specific) sedentary behaviours in hours/day, with a one-unit increase in the predictor.

Exponent b-values represent the proportional increase in (domain-specific) sedentary behaviours in hours/day, with a one-unit increase in the predictor.

### Moderating effects of gender, age and educational level

The significant moderating effects of gender, age and educational level on the associations between physical environmental neighbourhood factors and sedentary behaviours are presented in Table 4. Gender moderated five associations between objective physical environmental neighbourhood factors (three associations with safety, and two with aesthetics) and sedentary behaviours. Stratified analyses showed that perceived aesthetics was only related to transport-related sedentary behaviour in women. Women reported 0,17 hours (10,2 minutes) per day less transport-related sedentary behaviour per one-unit increase in perceived aesthetics. Age moderated seven associations, and educational level moderated four associations. Clear differences in direction were found for several associations in the stratified analyses on age and educational level, but none reached statistical significance.

**Table 4:** Significant moderating effects of gender, age and educational level on the associations between physical environmental neighbourhood factors and sedentary behaviours.

Moderator	Model	Association	b (S.E.) p^1^/ OR (95% C.I.)^2^	Stratified models	
				Groups	b (S.E.) p^1^/ OR (95% C.I.)^2^
**Gender**	Gaussian	**Safety (o)–Total SB**	**2.35 (0.94) 0.01**	Men	-0.25 (0.25) 0.31
				Women	-0.04 (0.14) 0.75
	Gamma	**Aesthetics (p)—Transport-related SB**	**-0.12 (0.05) 0.03**	Men	0.09 (0.06) 0.11
				Women	**-0.17 (0.05) <0.001**
	Gaussian	**Aesthetics (o)–Work-related SB**	**-1.86 (0.86) 0.03**	Men	0.36 (1.03) 0.73
				Women	-0.34 (0.95) 0.72
	Gamma	**Safety (o)–Computer time**	**1.41 (0.51) 0.01**	Men	0.17 (1.43) 0.91
				Women	0.08 (1.00) 0.94
	Gamma	**Safety (o)–Other leisure SB**	**1.66 (1.04, 2.64)**	Men	2.06 (0.53, 7.96)
				Women	0.93 (0.27, 3.14)
**Age**	Gaussian	**Safety (o)–Total SB**	**-3.58 (1.19) <0.01**	≤ 65 years	-1.14 (1.96) 0.56
				> 65 years	1.80 (3.08) 0.56
	Gaussian	**Functionality (o)–Total SB**	**-4.01 (0.94) <0.01**	≤ 65 years	0.81 (1.82) 0.66
				> 65 years	-4.12 (2.74) 0.13
	Gaussian	**Safety (p)–Work-related SB**	**2.32 (1.09) 0.03**	≤ 65 years	0.003 (0.16) 0.99
				> 65 years	2.49 (1.58) 0.12
	Gamma	**Aesthetics (o)–Other leisure SB**	**2.18 (1.16, 4.06)**	≤ 65 years	0.78 (0.50, 1.20)
				> 65 years	2.37 (0.65, 8.58)
	Gamma	**Destinations (o)–Other leisure SB**	**0.01 (0.00, 0.50)**	≤ 65 years	23.35 (0.88, 614.60)
				> 65 years	1.44 (0.00, 7601.85)
	Gamma	**Functionality (o)–Other leisure SB**	**0.45 (0.26, 0.77)**	≤ 65 years	1.00 (0.38, 2.62)
				> 65 years	1.17 (0.08, 16.62)
	Gamma	**Destinations (p)–Other leisure SB**	**1.20 (1.06, 1.35)**	≤ 65 years	0.96 (0.92, 1.01)
				> 65 years	1.09 (0.97, 1.23)
**Education**	Gamma	**Safety (p)–Television time**	**0.34 (0.14) 0.02**	Lower	-0.26 (0.16) 0.10
				Higher	0.05 (0.10) 0.60
	Gamma	**Safety (o)–Other leisure SB**	**1.85 (1.14, 3.00)**	Lower	1.79 (0.52, 6.15)
				Higher	1.18 (0.32, 4.28)
	Gamma	**Functionality (o)–Other leisure SB**	**1.72 (1.09, 2.71)**	Lower	0.61 (0.21, 1.82)
				Higher	0.93 (0.27, 3.25)
	Gamma	**Aesthetics (p)–Other leisure SB**	**1.10 (1.01, 1.19)**	Lower	0.92 (0.84, 1.01)
				Higher	1.02 (0.95, 1.09)

o = objectively measured, p = perceived

Significant values are indicated in bold.

OR = odds ratio, 95% C.I. = confidence interval at 95%, S.E. = standard error

^1^ b (S.E.) and p are reported for results of the Gaussian and Gamma (identity link) models.

^2^ OR (95% C.I.) is reported for results of the Logistic and Gamma (log link) models.

All b-values represent the increase in (domain-specific) sedentary behaviours in hours/day, with a one-unit increase in the predictor.

Exponent b-values represent the proportional increase in (domain-specific) sedentary behaviours in hours/day, with a one-unit increase in the predictor.

## Discussion

The first aim of the current study was to examine associations between physical environmental neighbourhood factors and sedentary behaviours among European adults. Both perceived and objectively measured aesthetics and destinations were found to be associated with domain-specific sedentary behaviours (work-related and other leisure-time sedentary behaviour), suggesting that physical environmental neighbourhood factors may play a role in determining sedentary behaviours. Although, the associations differed between perceived and objectively measured neighbourhood factors, it is noteworthy that only the constructs aesthetics and destinations were related to sedentary behaviours. As hypothesized, the direction of the associations was in the way that living in an attractive neighbourhood with many local facilities was associated with less leisure-time sedentary behaviour, and more work-related sedentary behaviour.

Regarding the objectively measured physical environmental factors, two significant associations were found. Both associations are difficult to compare with previous results, as, to our knowledge, no previous studies have examined these associations. Firstly, objectively measured aesthetics correlated negatively with the odds of engaging in leisure-time sedentary behaviour. Leisure-time sedentary behaviour comprises activities such as reading, socializing and going to a movie [45]. The majority of these activities take place indoors. It might be that, as expected, residents from attractive neighbourhoods are less likely to spend time indoors, and therefore less likely to spend time on leisure-time sedentary behaviour. Secondly, objectively measured destinations correlated positively with work-related sedentary behaviour. We expected that this would be the case due to the compensation mechanism [41,42]. More specifically, we expected that residents of neighbourhoods with many local facilities would be less likely to be sedentary for transport and during leisure-time activities, and that these lower levels would be compensated by being more sedentary at work. However, as no associations were found between the number of objectively measured destinations and transport-related or leisure-time sedentary behaviour, previous mentioned compensation mechanism cannot explain the association between objectively measured destinations and work-related sedentary behaviour. Consequently, more research is needed to confirm and clarify these potential associations.

Three significant associations with perceived physical environmental factors were observed. Although none of these associations correspond to the ones found for objectively measured physical environmental factors, it should be noted that, also here, significant associations were only found for aesthetics and destinations. Living in a neighbourhood with better perceived aesthetics was associated with more work-related and total sedentary behaviour. Again, our hypothesis was that residents from attractive neighbourhoods would be more sedentary at work to compensate lower levels of leisure-time and transport-related sedentary behaviour. However, no associations were found between perceived aesthetics, and leisure-time or transport-related sedentary behaviour. Alternatively, it may be that the association is due to residential self-selection. Previous studies have shown that work-related sedentary behaviour is positively associated with educational attainment [15,56] and household income [56], which are both proxies for SES. High SES adults may thus be more likely to choose to live in an attractive neighbourhood. Furthermore, our results showed that residents from neighbourhoods with more perceived destinations were less likely to have spent time sedentary during other leisure activities. This finding is in line with previously reported results [24,57], and adds to the evidence for a negative association between perceived destinations and leisure-time sedentary behaviour.

None of the objectively measured or perceived physical environmental neighbourhood factors we examined contributed to transport-related sedentary behaviour, television time or computer time. Since both television time and computer time during leisure usually take place at home, it seems likely that home environmental factors are more important than neighbourhood environmental factors for those two behaviours. In the review of Kaushal and Rhodes [18], the importance of home environmental factors to explain sedentary behaviour was emphasised, as this review showed that both the quantity and the location of televisions positively correlated with sedentary behaviour. However, the absence of significant associations for transport-related sedentary behaviour is more surprising. Nevertheless, previous research has also failed to detect consistent relationships between physical environmental neighbourhood factors and transport-related sedentary behaviour. Whereas some studies did find significant associations [25,54,58], others did not find significant associations [27]. A possible explanation, suggested by Koohsari et al. [23], was the fact that transport-related sedentary behaviour largely happens outside one’s neighbourhood. Nevertheless, more research is needed to confirm this explanation. Furthermore, no associations have been found with safety and functionality. Most previous studies also failed to find associations with safety and functionality [12,23]. In the reviews of Koohsari et al. [23], and O’Donoghue et al. [12], 75% of the studies reported non-significant associations between safety and sedentary behaviours, and approximately 70% of the studies reported non-significant associations between route-related attributes, such as traffic, connectivity and pedestrian infrastructure, and sedentary behaviours.

The second study aim was to test the moderating role of gender, age and educational level on the previously discussed associations. As expected, all three socio-demographic factors moderated different associations. Gender moderated associations with safety and aesthetics, indicating that the association of these two factors with sedentary behaviours differs between men and women. Age and educational level moderated associations with safety, aesthetics, destinations and functionality. However, the associations were non-significant and in different directions so no clear pattern could be identified, preventing us from drawing firm conclusions. Of the stratified analyses, only one significant association could be identified, probably due to a power issues. This significant association showed that women living in neighbourhoods with higher perceived aesthetics reported less transport-related sedentary behaviour compared to women living in neighbourhoods with lower perceived aesthetics. This does not correspond with the findings of Van Dyck et al. [25], who did not find a significant interaction effect of gender on the association between perceived aesthetics and motorized transport. The discrepancy of those study results may be due to differences in population characteristics.

There are some strengths and limitations that deserve attention. A first strength is that we included different domains of sedentary behaviour (i.e. transport, occupation and leisure-time) in relation with neighbourhood environmental characteristics. Previous studies examining the relation with neighbourhood environmental characteristics have mainly focused either on total sedentary behaviour or on just one domain of sedentary behaviour (e.g. television time). Furthermore, we examined both the perceived and objectively measured physical environment. This is of added value as previous studies have shown a clear mismatch between these two ways to measure the environment [32–35]. For the objective assessment of the physical environment, we used an instrument that was specifically designed for our project, and that had shown good validity and reliability. A final strength is the large study sample of more than 5,000 adults. This ensured sufficient statistical power to conduct generalized linear mixed models.

The most important limitation is the cross-sectional study design, precluding determination of causal relationships. Other limitations include, first, the self-reported data on behaviours, which may be subject to social desirability and recall biases. Second, the use of constructs to represent neighbourhood characteristics, which resulted in loss of information. Third, the lack of qualitative data on perceived physical environmental factors, such as the cost and maintenance of public transport facilities. And finally the low response rate of around 10%, which creates a potential risk of selection bias [59] (although the study sample seems representative of the adult population of Europe in terms of socio-demographic and behavioural characteristics [44]. There is a good representation of men (44%) and women (56%), lower (46.4%) and higher (53.6%) educated individuals as well as younger (from age 18 years) and older (up to age 109 years) adults [44]). Possible reasons for the low response rate include first the oversampling of low SES residents. Low SES residents have been shown to be less likely to participate in a health survey [60]. However, as we aimed to have a heterogeneous sample with as many low SES residents as high SES residents, we decided to oversample the former, which is likely to have led to a lower overall response rate. Secondly, with regard to the absence of an upper age limit, we know that there may be attrition in surveys where older people are less likely to be able to complete a survey due to, for example, limited cognitive function, or vision impairment [61]. In addition, the questionnaire was mainly administered online. Previous studies have indicated that Internet use drops off significantly after the age of 75 [62], also potentially contributing to a lower response rate. Thirdly, the survey was relatively long. Participants spend on average (SD) 25.1 (12.4) minutes to complete the questionnaire, which contained 50 key questions on 30 pages. Finally, we recognize that, in an era of frequent opinion polls and market research, people may react to what they perceive as over-surveying (i.e. become fed up with surveys). Although each of these factors, on their own, may not have had a large impact, they all act to reduce the response rate so, in combination, the effect may be appreciable.

## Conclusion

The results of the present study provide preliminary evidence for an association between physical environmental neighbourhood factors and domain-specific sedentary behaviours in adults. Associations were found between perceived and objectively measured aesthetics and destinations, and work-related and leisure-time sedentary behaviours. Although both measurement methods revealed associations with the same environmental factors (i.e. aesthetics and destinations), it should be noted that the directions of the associations differ. These differences make it difficult to draw meaningful practical implications. Moreover, the results need to be confirmed by other, preferably longitudinal, studies, before recommendations can be made. Furthermore, as we only studies urban areas, our results do not apply to rural residents. Future studies should include both urban and rural areas to determine if associations differ by residential area. Moderation analyses showed interactions of age, gender and educational level in the neighbourhood environment—sedentary behaviour relationship, suggesting that the contribution of one’s neighbourhood environment to sedentary behaviours varies across different subgroups.

## Abbreviations

95% CI: Confidence interval at 95%
SES: Socioeconomic status
BMI: Body mass index
Adj. OR: Adjusted odds ratio
